# A framework for quantifying the extent of impact to plants from linear construction

**DOI:** 10.1038/s41598-017-02443-3

**Published:** 2017-05-30

**Authors:** Jun Xiao, Peng Shi, Ya-Feng Wang, Yang Yu, Lei Yang

**Affiliations:** 1 Key Laboratory of Agro-ecological Processes in Subtropical Region, Institute of Subtropical Agriculture, Chinese Academy of Sciences, Changsha, 410125 China; 2 State Key Laboratory of Urban and Regional Ecology, Research Center for Eco-Environmental Sciences, Chinese Academy of Sciences, Beijing, 100085 China; 3 Key Laboratory of Northwest Water Resources and Environment Ecology of Ministry of Education, Xi’an University of Technology, Xi’ an, 710048 China; 4 Department of Sediment Research, China Institute of Water Resources and Hydropower Research, Beijing, 100048 China

## Abstract

We present a novel framework that accurately evaluates the extent of a linear project’s effect from the variability of the structure of the plant community while avoiding interference caused by pioneer species and invasive species. This framework was based on the change of dominant species in the plant community affected by construction. TWINSPAN classification and variation of the integrated importance value (IIV) of each plant species group were used to characterize the process of change in the structure of the plant community. Indicator species group and its inflection point were defined and used to judge the extent of the effects of pipelines. Our findings revealed that dominant species in the working area of the pipeline construction were different from the original plant communities. With the disturbance decreased, the composition and structure of the plant communities gradually changed. We considered the outer limit of the area affected by the construction to be the first area in which the plant community reached a steady state and was similar to the original community. The framework could be used in the post eco-environment impact assessment of linear construction to estimate the intensity of disturbance and recovery condition.

## Introduction

Oil and gas pipeline construction is an important type of linear project. Pipelines have been widely used in recent years due to their obvious advantages, e.g., low cost and consumption, high efficiency, and large transportation volume^1, 2^. Although pipeline construction plays an important role in energy transportation and social improvement, it simultaneously disturbs the surrounding area and causes several eco-environmental problems^3^. Surface vegetation is usually removed for ditch excavation and road paving that accompany pipeline installation^4^. The structure and properties of the soil (such as bulk density, pH, temperature, organic matter content and heavy metal content) change after soil compaction and backfilling, and these changes can result in habitat fragmentation^5–8^ and the edge effect^9^, along with an influx of invasive species^10^. Furthermore, the influences of pipelines are complicated and varied because these large-scale projects usually span multiple regions and ecosystems. The focus of these eco-environmental problems varies in different ecosystems^11–13^ that have gradually gained the attention of the public. Eco-environmental impact assessment and management of these problems is becoming a common concern in middle- or large-scale development projects^14, 15^.

Estimating the scope and extent of the effect is important for ecological restoration and environment management, especially for eco-environmental impact assessments and vegetation restoration. Several studies have examined the effects of pipeline construction on vegetation, soil, and landscape patterns^16–20^. However, such studies usually focus on specific ecosystems and often use regular analysis methods that require monitoring of the variability of the soil and/or vegetation and need substantial amounts of time. Thus, it is necessary to develop a universal method that can quickly estimate the effect intensity.

Because plants are removed during the construction stage and then natural recovery or artificial restoration is carried out during the operational stage^21^, the plant community is adversely affected by the disturbance of pipeline construction^22^. The community structure of the working area greatly differs from that of the original environment^10^ and the areas surrounding the pipeline are also affected by human disturbance, albeit at a lower intensity. This reduction in intensity is reflected in the alteration of the plant community and is relatively easily observed^23^. Hence, it is possible and meaningful to determine the effect of pipeline construction by analyzing the variability of the surrounding plant community.

In China, several large oil and gas pipeline construction projects are in operation, such as the West-East Gas Pipelines (WEGPs, including WEGP I and WEGP II), the Western Crude Oil Pipeline (WCOP), and the China-Russia crude oil pipeline project^23, 24^. In this study, we chose an area in northwest China through which both the WEGPs and WCOP pass and investigated the vegetation conditions along the area of construction. We used comparative analysis and TWINSPAN classification to classify plants into groups and identify the changes in the plant community structure after construction. Finally, we developed and verified a framework that can estimate the intensity and area of the pipeline effect. As a type of linear construction, pipeline construction shares the same characteristics with other linear construction such as roads, railways, and highways^25^. Therefore, this framework could be used in estimating the effect of linear constructions in the eco-environmental impact post-project-assessment (EIPPA) and supplying data support for an eco-environmental restoration method.

The objectives of this study were to (1) analyze the response of the plant community being disturbed by pipeline construction and (2) propose a theoretical paradigm that could indicate the effect scope of the construction by this response.

## Results

### Plant species classification

**Figure 1:**
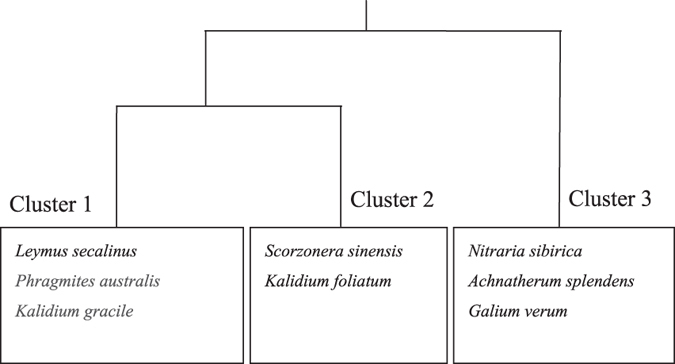
Results of cluster analysis of plant species by TWINSPAN.

**Figure 2:**
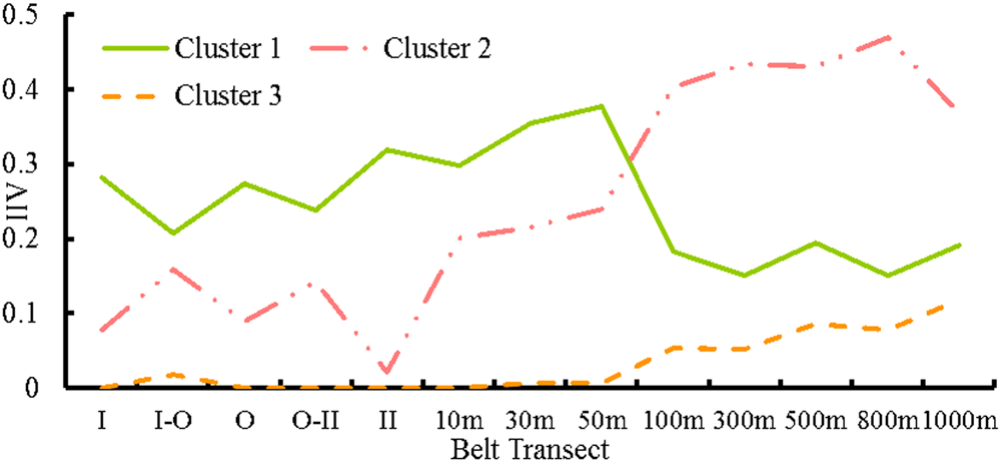
Changes in IIVs of the three clusters in the belt transects in Study Area S1.

The eight plant species found across the sampling plots in the Study Area S1 (Table 1) grouped into three clusters by TWINSPAN by their importance values (Fig. 1). *Leymus secalinus* (Georgi), *Phragmites australis* (Cav.) Trin. ex Steud., and *Kalidium gracile* Fenzl were clustered in the same group (Cluster 1) and the second group (Cluster 2) included *Scorzonera sinensis* Lipsch. et Krasch. ex Lipsch. and *Kalidium foliatum* (Pall.) Moq. The third group (Cluster 3) included *Nitraria sibirica* Pall., *Achnatherum splendens*, and *Galium verum* L. Different clusters had different processes of their position changing in the community (Fig. 2). Plants in Cluster 1 were found across the belt transects but their integrated importance values peaked within 50 m of the pipelines. *P. australis*, one of the three plants in Cluster 1, was dominant in the pipeline area. A huge change of dominant position was found from 50-m to 100-m belt transect. Cluster 2 Plants took the dominant place of plants of Cluster 1 in the community. They were also found in all belt transects but peaked 100–1000 m away from the pipeline area. *S. sinensis* and *K. foliatum* were relatively rare in the pipeline area. Cluster 3 plants were rare in all belt transects but were typically absent less than 30 m from the pipelines.

**Table 1:** Importance value of each species and IIV of each Cluster in the belt transects.

Cluster	Cluster 1				Cluster 2			Cluster 3			
Belt Transect	*Kalidium gracile*	*Leymus secalinus*	*Phragmites australis*	IIV	*Kalidium foliatum*	*Scorzonera sinensis*	IIV	*Achnatherum splendens*	*Galium verum*	*Nitraria sibirica*	IIV
I	0.133	0.089	0.622	0.281	0.081	0.074	0.078	0	0	0	0
I-O	0.085	0.303	0.236	0.208	0.085	0.234	0.16	0	0.057	0	0.019
Oil	0.206	0.097	0.518	0.274	0.097	0.082	0.09	0	0	0	0
O-II	0.095	0.214	0.406	0.238	0.077	0.209	0.143	0	0	0	0
II	0.442	0.054	0.462	0.319	0.042	0	0.021	0	0	0	0
10 m	0.076	0.337	0.482	0.298	0.122	0.280	0.201	0	0	0	0
30 m	0.168	0.314	0.581	0.354	0.126	0.306	0.216	0	0.022	0	0.007
50 m	0.182	0.391	0.558	0.377	0.228	0.250	0.239	0	0.020	0	0.007
100 m	0.153	0.134	0.263	0.183	0.384	0.422	0.403	0.035	0.060	0.068	0.054
300 m	0.179	0.094	0.18	0.151	0.491	0.379	0.435	0.035	0.059	0.064	0.053
500 m	0.180	0.275	0.127	0.194	0.477	0.384	0.431	0.094	0.113	0.055	0.087
800 m	0.183	0.180	0.092	0.152	0.540	0.400	0.47	0.096	0.081	0.058	0.078
1000 m	0.245	0.241	0.089	0.192	0.382	0.355	0.369	0.180	0.113	0.062	0.118

Note: Pipeline area, the area in which pipeline was laid, encompasses belt transects I, Oil and II. I-O and O-II are the belt transects set in the middle of WEGP I and WCOP, WCOP and WEGP II, respectively. 10 m, 30 m, 50 m, 100 m, 300 m, 500 m, 800 m, 1000 m are the belt transects outside10 m, 30 m, 50 m, 100 m, 300 m, 500 m, 800 m, 1000 m of the northern edge of the pipeline area of WEGP II.

**Figure 3:**
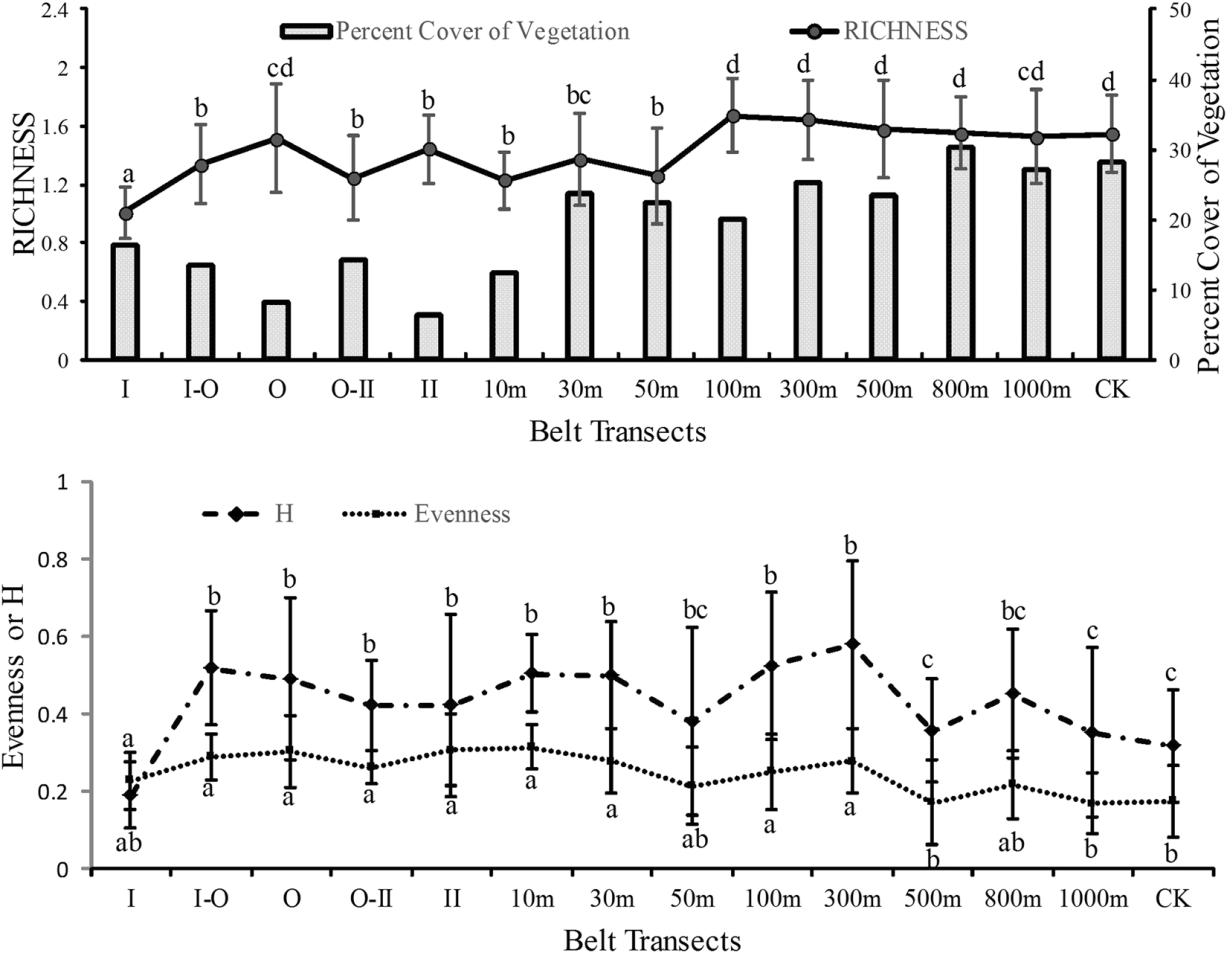
Changes in RICHNESS, Percent Cover of Vegetation, H and Evenness of the plant communities in the belt transects. CK was the Control Belt Transect which was at least 2 km north from the pipeline area and was rarely affected by human disturbance. For each index, bars marked with the same letter are not significantly different from each other at the p = 0.05 level. The bars of H are marked letters below, while the bars of Evenness are marked above. The bars showed +/− SE.

The vegetation structure significantly differed between the pipeline area and the surrounding area (Fig. 3). The Richness in the pipeline area as well as 10-m and 50-m belt transects, was low, whereas it got to the highest value in the 100-m belt transect. From 100-m to CK (sampling plots which were 2000 m away from the edge of northernmost pipeline area were selected as CK), Richness was stable and significantly higher than the pipeline area to 50-m belt transect. The changes of Percent Cover of Vegetation reflected the intensity of disturbance. Low intensity disturbance still affected the ecosystem after construction finished, and vegetation conditions of the farther area from the pipeline area were less disturbed than the nearer area. In 10-m belt transect, a companion road (lane) was formed by running over land by the construction vehicles and used for people working on the pipelines’ routine maintenance and for patrol. These low frequency disturbances still affected the 10-m belt transect, and kept the effect on the surrounding area. The changing of H (Shannon-Wiener diversity index) showed that the diversity of the pipeline area was higher than the control. Besides, there were relatively high values of H in the 100-m and 300-m belt transects. This indicated that diversity increased in the areas closed to working area of the pipeline. Evenness was relatively stable in the community from in the belt transects. Evenness of CK was lower than that of affected belt transects.

### Correlations between IIVs and vegetation clusters

**Figure 4:**
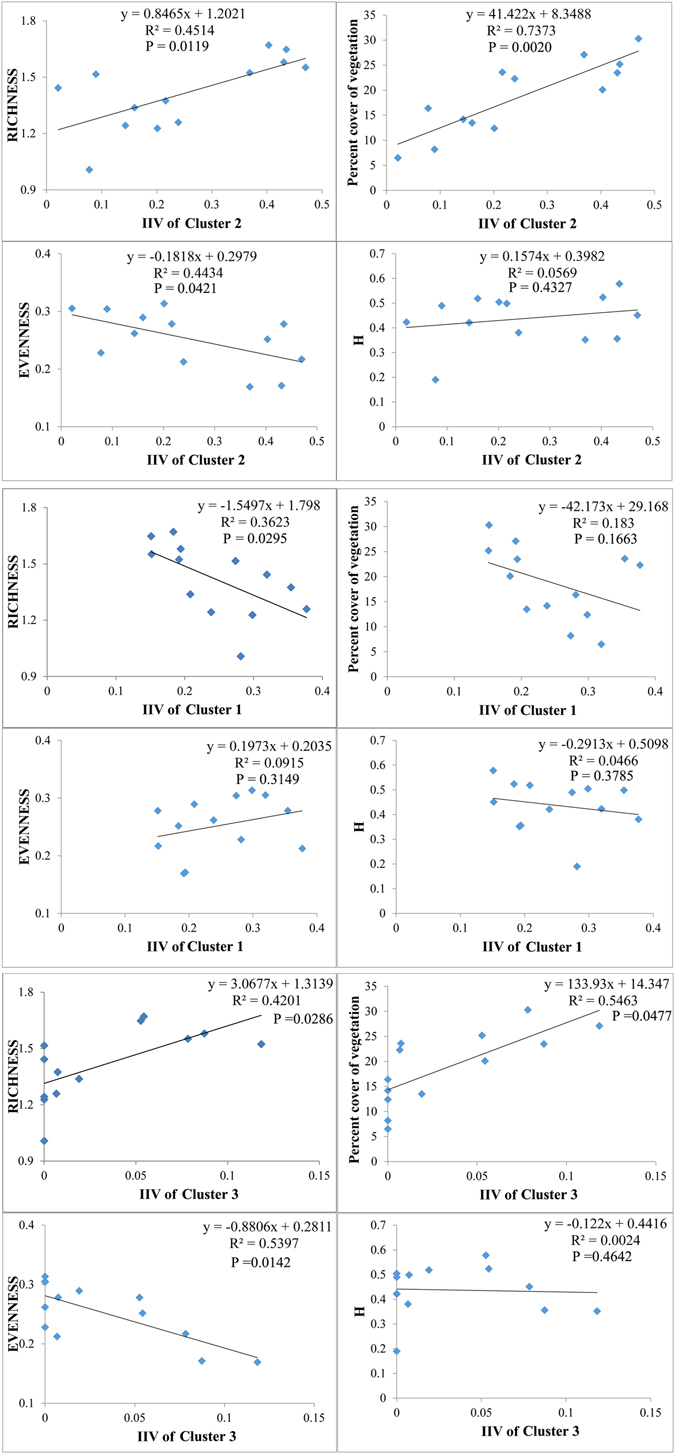
Correlations between the vegetation indices and IIVs of the plants species of three Clusters.

Correlations between the IIV of Cluster 2 and Percent Cover of Vegetation, Richness, Evenness, and H showed the IIV of Cluster 2 had a significantly positive relationship to the Percent Cover of Vegetation of the plant community (p < 0.01) and Richness and Evenness (p < 0.05) (Table 2), but little relationship with H (Fig. 4). Thus, the IIV of Cluster 2 mainly reflected the Percent Cover of Vegetation of the plant community and partly represented the richness. Cluster 1 was inadequate to be the indicator species which could be used to reflect the response of plant community to the distance, because IIVs of Cluster 1 did not significantly correlate with the community indices except Richness. IIVs of Clusters 3 significantly correlated with Percent Cover of Vegetation coverage and Evenness. However, five belt transects were lacking the value of IIVs of Cluster 3. To avoid the randomness in the comparison analysis, Cluster 3 was not recommended for tracking recovery because it contains less abundant species that could be missing from undisturbed or recovered sites due to their low abundance.

**Table 2:** Correlation between IIVs of Clusters and vegetation indices.

	n	Mean	Variance	p Value		
				IIV of Cluster 1	IIV of Cluster 2	IIV of Cluster 3
RICHNESS	78	1.41	0.31	0.0295	0.0119	0.0286
Percent Cover of Vegetation	78	18.72	12.34	0.1663	0.0020	0.0477
EVENNESS	78	0.25	0.07	0.3149	0.0421	0.0142
H	78	0.44	0.16	0.3785	0.4327	0.4642

### Effect on different plant classifications and its theoretical framework

Considering the changes in the IIVs of the three clusters of plants, we believed that the plants in Cluster 1 were becoming more abundant and therefore the dominant species in the plant community after human disturbance; the plants in Cluster 2 were those whose IIVs were reduced and then lost their original dominant position in the plant community because of the disturbance; and the Cluster 3 plants were sensitive to disturbance and thus grew poorly. They were also accompanying species (that were common in the community but hardly took the dominance of the community) in the original ecosystems.

*P. australis*, *L. secalinus*, and *K. gracile*, which could be considered pioneer species in the vegetation recovery, occupied the main ecological niche space in this area^26^ in the two years after the constructions was completed^26^. These species had high resistance and dispersal ability and could acclimatize to the new environmental condition. Compared with other species, their growth was less negatively affected by the disturbance brought about by the pipeline construction. They recovered quickly and took the dominant position in the community.

**Figure 5:**
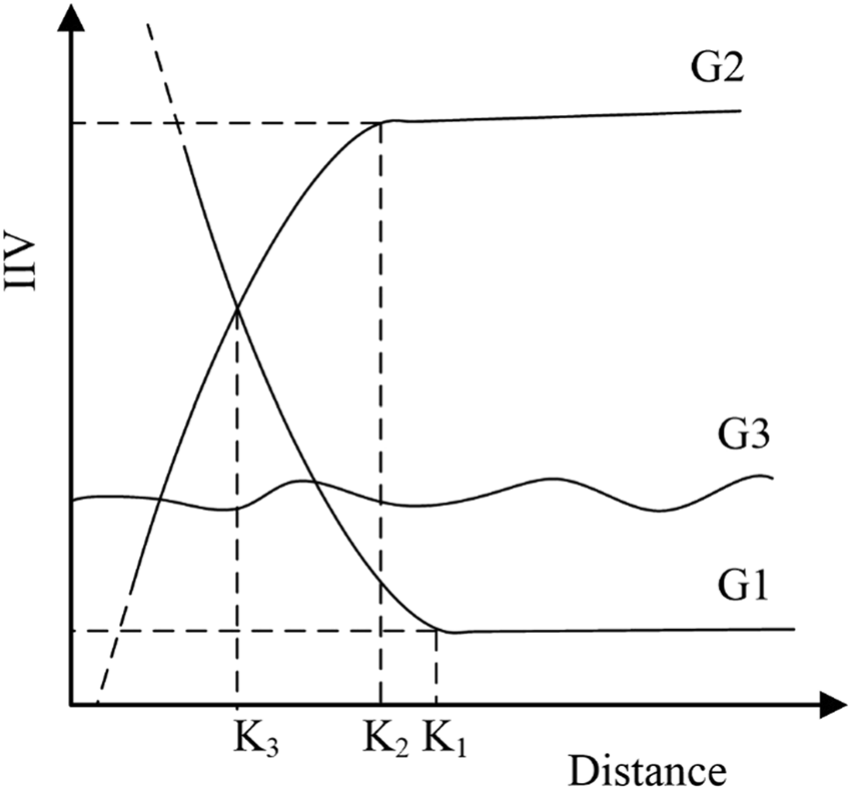
Theoretical paradigm for assessing the effect of pipeline project construction on changes in the structure of the surrounding plant community. Distance represents the distance from the pipeline construction. G1 represents the plant species with low or non-existent presence in the original ecological communities, but became dominant plants after being affected by the construction and incidental factors. G2 represents the plant species with dominant positions in the original ecological communities but whose IIV decreased in the area of pipeline construction. G3 represents the remaining plants not classified as G2 or G3.

From the changes in IIVs of the three clusters of plants, we can assume that the effects of the pipeline construction or other linear projects on the surrounding plant communities can be classified into three types, called Group 1, Group 2, and Group 3 (G1, G2, and G3, respectively) (Fig. 5). In Study Area S1, Cluster 1 to Cluster 3 are equated to G1 to G3, respectively, whereas clusters are usually the sub-classifications of groups. We define groups as these classifications that are sorted at the first and second break in the calculating process, while clusters are the result of classification. The G1 and G2 curves indicate a drastic change in the trajectory of the IIVs of Cluster 1 and Cluster 2. G1 represents the plant species that were rare or even non-existent in the original ecological community but that occupied the dominant position after the construction disturbance. G2 represents the plant species whose IIVs sharply decreased and then lost their original dominant position in the plant community after the disturbance. G3 represents the remaining plants that did not belong to either G1 or G2. The trajectory of IIVs in Cluster 3 can take various forms, but is different from those of G1 and G2. When the IIV trajectories of G1 and G2 become smooth and steady, the inflexion points of K1 and K2 may be regarded as the boundary of the effect extent for those two clusters of plant species. Because G2 represented the originally dominant plants, the effect extent of pipeline construction or other disturbances of the ecological environment can be determined by K2. K3 marks the position of G1 and G2 at which the plant community changes. The structure of the community is greatly altering in the area marked by K3, and the edge effect is obvious because of high diversity and intense interspecific competition. This area is a key belt transect that requires more attention during environmental management and artificial ecosystem recovery. Then G1 to G3 are determined by the trajectory of the IIVs.

Vegetation and the plant community have been seriously affected in the construction period^19, 27^. As the pipeline begins operation, vegetation can gradually recover with the decrease in disturbance^28, 29^. Due to the variability of effect intensity at different stages of construction, K1, K2, and K3 change dynamically. They increase during the construction period and decrease once the pipeline becomes operational. After the intensity of disturbance levels off, the value of K can stabilize. The effect scope and extent (K2) can be accurately obtained using this method because only species that are dominant in the original environment and negatively affected remain for analysis. However, alien species, positively affected species, species with little response, and some negatively affected species are excluded. Presently, the stable range of K must be determined by empirical measurement in this framework, and this needs to be improved in subsequent work.

### Verification of the theoretical framework in a different region

According to the characteristics of the effect of pipeline construction on the succession characteristics of the plant communities, we proposed a theoretical framework for estimating the effect extent by screening the indicator species and calculating the value of IIV. Although this theoretical model showed a positive correlation with some vegetation indices, it was based on research work in a specific research area and its applicability needed to be verified. Therefore, we used this proposed theoretical framework to assess its applicability in the Study Area S2.

**Figure 6:**
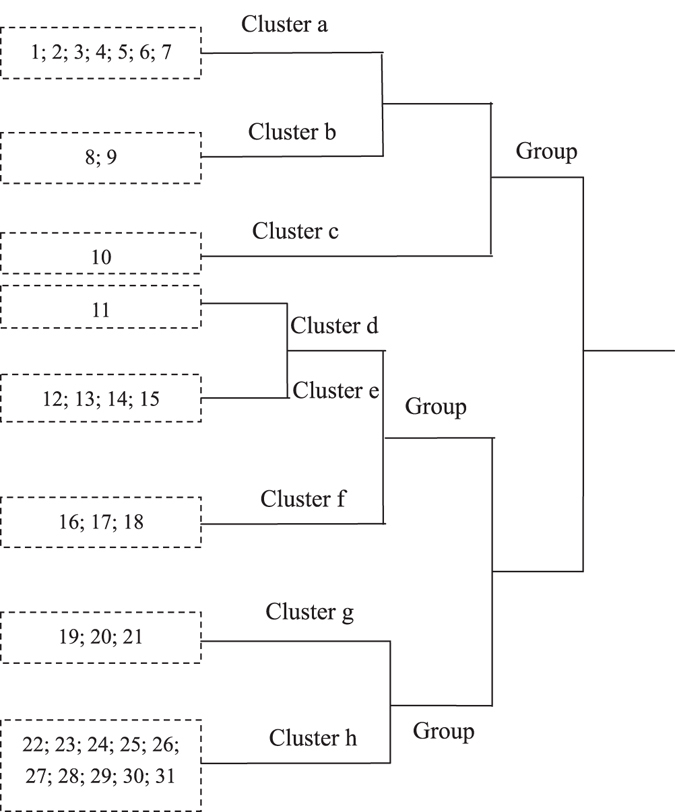
Results of TWINSPAN for the data from the plots in S2. Note: Species abbreviations are 1: *Cosmos bipinnata* Cav.; 2: *Heteropappus altaicus* (Willd) Novopokr; 3: *Caragana Korshinskii* Kom; 4: *Neotorularia humilis* (C. A. Meyer) Hedge & J. Léonard; 5: *Gueldenstaedtia verna* (Georgi) Boriss.; 6: *Polygala wattersii* Hance; 7: *Convolvulus ammannii* Desr.; 8: *Suaeda glauca* (Bunge) Bunge; 9: *Potentilla bifurca* L., 10: *Leontopodium leontopodioides* (Willd.) Beauv.; 11: *Lespedeza davurica* (Laxm.) Schindl., 12: *Allium mongolicum* Regel.; 13: *Setaria viridis* (L.) Beauv.; 14: *Limonium bicolor* (Bunge) Kuntze; 15: *Pseudognaphalium affine* (D. Don) Anderberg; 16: *Stenosolenium saxatile* (Pallas) Turczaninow; 17: *Corispermum mongolicum* Iljin; 18: *Hyoscyamus niger* L.; 19: *Gueldenstaedtia stenophylla* Bunge; 20: *Caragana brachypoda* Pojark.; 21: *Artemisia frigida* Willd.; 22: *Digitaria sanguinalis* (L.) Scop.; 23: *Stipa tianschanica* Roshev. var. *gobica* (Roshev.) P.C.Kuo et Y.H.Sun; 24: *Artemisia capillaris* Thunb.; 25: *Peganum harmala* L.; 26: *Astragalus discolor* Bunge ex Maxim. in Bull.; 27: *Tribulus terrester* L.; 28: *Cleistogenes squarrosa* (Trin.) Keng; 29: *Achnatherum splendens* (Trin.) Nevski; 30: *Cleistogenes caespitosa* Keng; 31: *Euphorbia humifusa* Willd. Group 1 to Group 3 would be confirmed in the next step. Therefore, G1 to G3 were not labeled in this figure.

**Figure 7:**
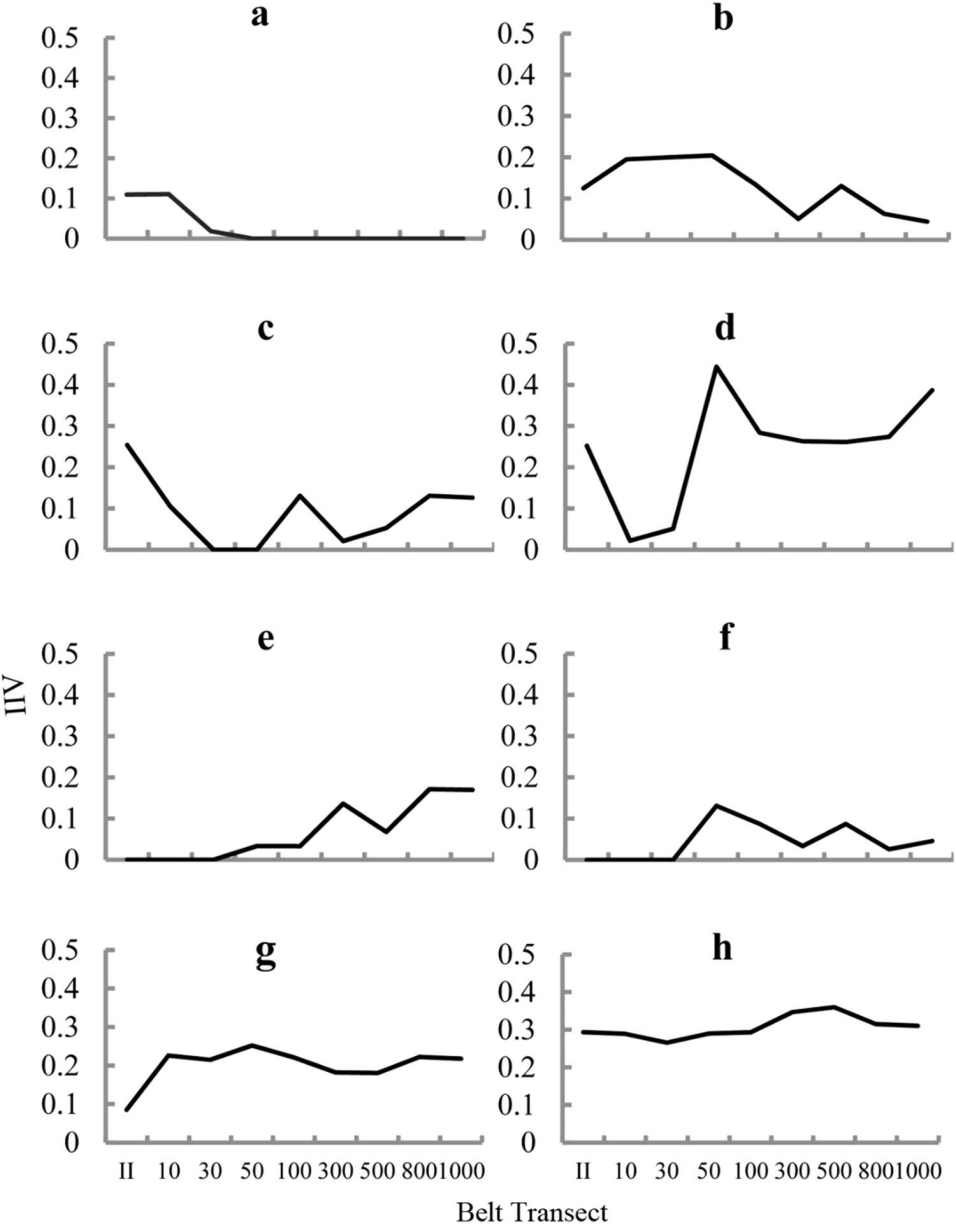
Analyses for the IIVs of the different groups of plants. Clusters a-c belonged in the G1. They were the group of species that was less affected near the construction. Clusters d-f were clustered in G2. They were dominant position in the original plant community but obviously affected by the construction. Clusters g-h were common in all the belt transect.

After partial accidental species were excluded from the survey, TWINSPAN was used to classify the plant species in S2 (Fig. 6). More sub-classifications were added because there are more plant species in this region. The variation of the IIVs of the eight clusters in the belt transects was shown in Fig. 7, which reflects the position of the different plant species in the community and their spatial relationship with pipeline construction. According to the theoretical framework, Clusters a, b, and c belong to G1. Cluster a was the typical indicator species, which indicated the effect scope of the introduced species. Clusters d to f belonged to the G2 plant classification, and Clusters e, and f were typical classifications. Cluster d was assigned to the G2 because it was obviously affected in the 10–30-m range, which meant that it was sensitive to the companion road during the operational period. Clusters g and h represented G3, which were common in the local environment and were strongly resistant to the disturbance.

**Figure 8:**
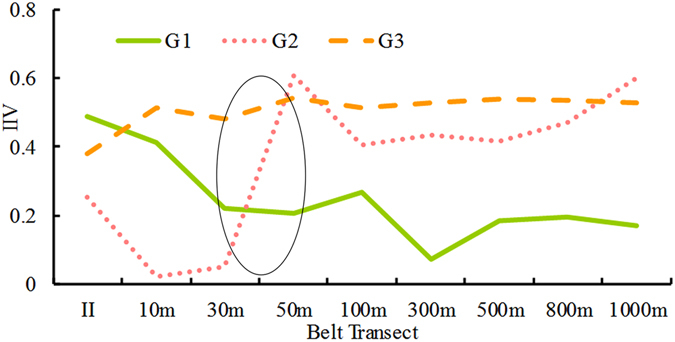
Changes in the IIVs of the three Clusters of plant species. The structure of plant communities in 30-m to 50-m belt transect area changed drastically, and became stable beyond 100-m belt transect.

The classification results were fitted to the theoretical framework illustrated in Fig. 8, which shows the variation in the IIVs across the different belt transects. G1 to G3 fairly indicated the response of vegetation to the construction compared with the IIV of the plant community in the original eco-environment. Then we could find out the extent of disturbance of WEGP II, which was the pipeline area to the 30-m belt transect. It was narrower than that of Study Area S1, proving that multiple constructions have a more intense effect on the eco-environment. This showed that the framework could generalize the rules and characteristics of the effect on the plant community of a different region. Therefore, determining the extent and scope of the effect through an indicator species appeared to be a rapid method that achieved high efficiency and accuracy.

## Discussion

### Effects of pipeline construction on the plant community

Pipeline construction had a strong influence on the vegetation communities located in the pipeline area. Even the IIVs of Cluster 1 were slightly lower in the working area than the first 50 m outside in S1. Vegetation patterns also were affected between the pipelines, but the disturbance was less than that in the pipeline area^30^. The main area of the plant communities affected by pipeline construction was from the pipeline area to the 100-m belt transect, whereas vegetation patterns recovered or maintained their original condition beyond the 100-m belt transect. From the 50-m to 100-m area, intense competition between species occurred and the dominant species in the plant community changed finally. In these areas, species richness and diversity were higher than the surrounding area, which showed the edge effect. The position of the species of Cluster 1 sharply declined in this region and richness as well as diversity was high, showed a clear edge effect^31–34^, thus indicating that this region is a transition region between the artificial corridor and the original natural environment^35^. This phenomenon could be explained by the intermediate disturbance hypothesis^36^. The effect of human disturbance such as routine maintaining and patrol brought by construction, lasted well after all construction completed. Because this belt transect was moderately affected, the moderate disturbances changed the community structure, and ultimately increased the biological diversity^37, 38^.

Pipeline construction had a significant influence on the surrounding plant community and its succession process. The plant community did not recover to its original condition after 3 years of natural recovery, and routine patrol and maintenance were brought into this area after construction completed, indicating that the pipeline construction still had a long-term ecological effect on the surroundings^23, 39^.

The IIV of Cluster 2 reflected the variability of vegetation coverage, richness and evenness, because of their significant correlations, which were the direct response of the ecological community structure to the pipeline. It was not significantly correlated with H, which reflected the diversity. Previous studies suggested that the diversity of plant species undergoes a high increase in the first year of the restoration succession^40^, which is not synchronized with the recovery of vegetation coverage^41^. It was supported by this study (Fig. 3).

Therefore, in Study Area S1, based on the variation of IIVs of G2, the pipeline area to the 50-m belt transect was the main affected area. An edge effect was clearly shown in the 50–100 m area. The plant community reached a steady state outside the 300-m belt transect. Hence, the 300-m area was considered as the K2 point, which indicates the extent of the disturbance brought by construction.

WEGP II was the only pipeline construction laid in S2 and was the latest one in S1. Because of the same belt transect setting, S1 and S2 could be reasonably compared. In S2, the 100-m area was considered as the K2 point, and the edge effect was shown in the 30–50 m area. This indicated that the effect of one pipeline being constructed was less than that of two or three, which could be explained as the accumulation effect by multiple pipelines^21^. The low intensity disturbance brought by construction still affected the vegetation recovery after construction was completed. This disturbance intensity would be increased when two pipeline constructions interacted in one area. It seemed that the plant community was sensitive to this increased disturbance. Thus, the structure of plant community was reasonable to represent the effect intensity of construction. Some studies reported multiple linear constructions, which would cause an accumulation effect on the eco-environment^21, 30, 42^. Therefore, the accumulation effect should be considered in the EIPPA.

### Comments about the framework

This framework is derived from a case study. Its main principle is based on the responsive structure of the plant community to the effect brought by construction to judge the effect of intensity. The change of structure is represented by the changes of IV of a group indicator species across the different distances of belt transects. The change of K can reflect the change of disturbance in both spatial and temporal scales. Moreover, K can indicate the accumulation effect of multiple linear constructions. Though G1 is less important than G2, it is still useful to indicate the effect scope of positively affected species. G1 and G2 can determine the K3 to reflect edge effect.

The belt transects setting in the farther area should be determined by the eco-environmental condition. Considering the areas nearer construction are more affected by construction, we could add the belt transects to improve the accuracy of the results. For instance, if a belt transect was added between 100-m and 300-m belt transect in Study Area S1, a more precise effect scope would be determined. On the other hand, some belt transects could be left out when they were obviously less effected by the target construction.

This framework could service as a guide to estimate the effect intensity of pipeline construction. It is theoretical, feasible, and could provide an approach to identifying environmental impact. Meanwhile, other linear constructions, such as roads and railways, could also be applied to this framework. As to other infrastructure, it could provide an option to estimate the intensity of the effects. This framework, however, is restricted by two main factors. First, the vegetation condition is most important. It is not fit for artificial ecosystem, such as urban ecosystem and farmlands. Second, distinguishing disturbances caused by different constructions is difficult. In this study, the two study areas were merely affected by the linear construction and its appurtenant work. There were no other types of disturbance or other construction. But some areas may be affected by the various kinds of influences caused by many other buildings. These influences make it hard to distinguish the effect intensity caused by the target construction. For these reasons, this framework is mainly oriented toward the linear construction. Whether it could be used in estimation of other constructions’ impact depends on the building environment of target infrastructure. The modification method for improving the feasibility of framework on the non-linear construction and complicated environment warrants further study.

## Conclusion

The construction of the pipelines had a significant effect on surrounding vegetation environment, and its influence on specific plant species can be properly reflected by the variability of the importance value in the plant communities. The effect scope and extent could be estimated per the different responses of the plants to the disturbance. Thus, a novel theoretical framework was proposed that can be used to assess the effects of pipeline construction and other linear construction on ecology and the environment, especially the composition and structure of the surrounding plant community. Under this framework, a new concept of G cluster of plant species was introduced that represented three different responses to construction by the plant species. Plant species could be divided into three types by TWINSPAN classification: species changing from rare or accompanying species in the work area to dominant species in the original environment (G2), species changing from the dominant species in the work area to accompanying species in the original environment (G1), and species not included in either G2 or G1 (G3). G2 was taken as indicator species, and the variation of its IIV was used to quickly judge the scope and strength of the pipeline construction effect. The developed framework could provide a convenient and relatively rapid method to increase efficiency and reduce the workload of post-eco-environmental impact assessment, ecological restoration, and environmental management when judging the scope of influence or finding key target of restoration. The applicability of this theoretical framework to different ecosystems and different types of construction needs to be verified or improved in future studies.

## Materials and Method

### Study Area

#### Desert Steppe in Guazhou

**Figure 9:**
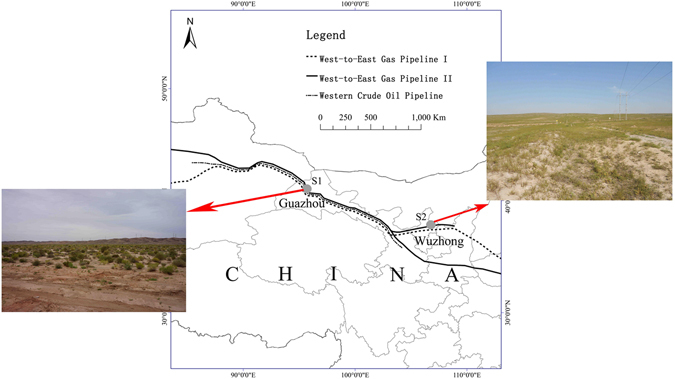
Location of the study area. This map was created using ArcGIS 10.2.2 software. There were three pipeline in the S1. The northernmost one was WEGP II, and the southernmost one was WEGP I. There was only one pipeline in S2, which was WEGP II.

Study Area S1 is in Anxi Arid Desert National Nature Reserve, which is in Guazhou county, Gansu province (Fig. 9). It is a transitional region between warm temperate zone and middle temperature zone with a mean annual temperature of 8.9 °C and a low mean annual precipitation of 48 mm (average 1951–2004). The steppe desert was formed because of shallow groundwater. Scarce species types are dispersed across the steppe desert, facilitating species classification and analysis. As an ecologically fragile area, it is sensitive to human disturbance, and the extent of the effect of pipeline construction is obvious and readily identified. These features help us to summarize the regular response of the plant community to the disturbance.

The West-East Gas Pipeline I (WEGP I, serviced in 2003), West-East Gas Pipeline II (WEGP II, serviced in 2013), and Western Crude Oil Pipeline (WCOP, serviced in 2006) pass parallel through this area from the northwest to the southeast. WCOP is in the middle, and WEGP I is on the south side of WCOP. The distance between the two pipelines is about 10 m. In the pipeline area, *P. australis* is now the dominant species, and *L. secalinus* and *K. foliatum* are the accompanying species, whereas *S. sinensis* and *K. foliatum* are the dominant species in the original surrounding area. Additionally, *Tamarix chinensis* Lour. is one of the few shrubs sparsely distributed in this area.

#### Desert Steppe in Wuzhong

Study Area S2 has more plant species and a lower intensity of human disturbance than S1, and was thus selected to verify the hypothesis of the framework formulated in the text. This area is in the south of Hongsibao county, Wuzhong city (Fig. 9). It has a typical continental monsoon climate with mean annual temperature of 9.4 °C and mean annual precipitation of 258 mm (average 1981–2010). Winters are cold and dry, and most rainfall occurs during the summer. The soil is aeolian sandy soil with poor fertility. The land type is grassland and desert steppe, which is fragile and prone to degeneration. WEGP II passes through from west to east. The working area was about 8 m wide, and a companion road was formed by workers walking along the pipeline.

### Setting of belt transects and data investigation

**Figure 10:**
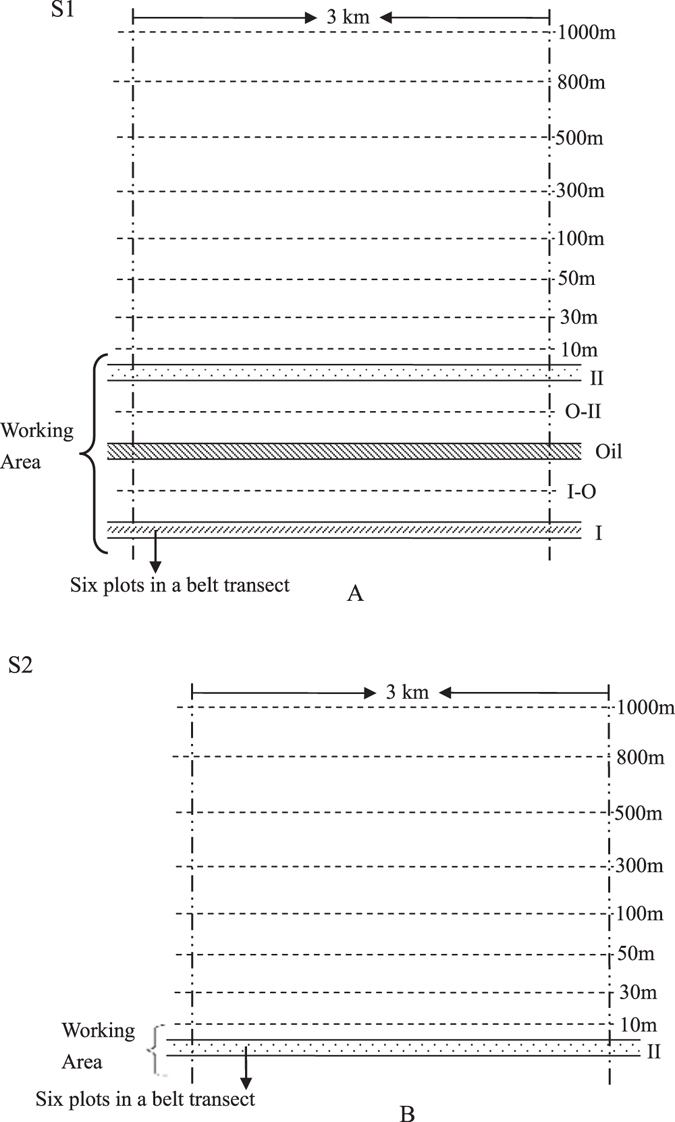
Design of the belt transects in S1 and S2. I, Oil, and II represent the belt transects set in the pipeline area of WEGP I, WCOP, and WEGP II, respectively; I-O and O-II are the belt transects between WEGP I and WCOP, and WCOP and WEGP II, respectively. 10-m, 30-m, 50-m, 100-m, 300-m, 500-m, 800-m, 1000-m belt transects were set on the north of pipeline area because there was a railway about 2 km to the south of the pipeline area in the S1 and a national road in the S2. The north part of the pipeline area was not affected by other construction and was natural environment before pipeline construction.

**Figure 11:**
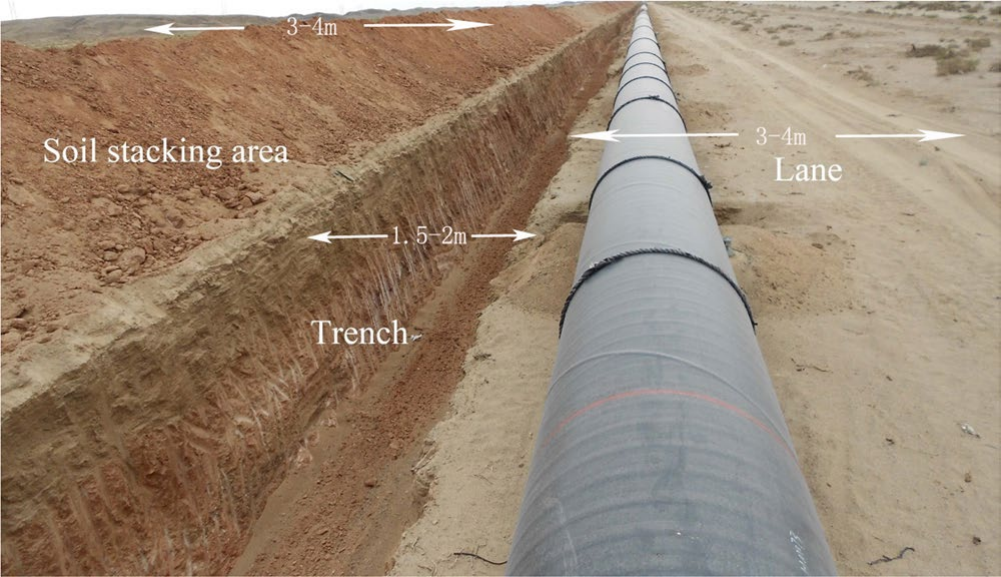
Working area of the pipeline. The photograph was taken in Guazhou county during the construction of WEGP II. When construction was completed, the trench was renamed pipeline area, and soil stacking area began natural recovery. The lane was usually kept and used as a companion road. The trench was about 1.5–2 m wide. The Lane and Soil stacking area were both 3–4 m wide.

In S1, to avoid the disturbance of railway to the south, belt transects were delineated to the north of pipeline area of WEGP I. The centers of the belt transects placed 10, 30, 50, 100, 300, 500, 800 and 1000 m from the edge of the northernmost pipeline area. Belt transects were also set in each of three pipeline areas and between each set of neighboring pipelines. Each belt transect was about 3 km in length. Then, sampling plots of 2 × 2 m were created (Fig. 10A). At each belt transect, six sampling plots were randomly selected for vegetation investigation resulting in 13 belt transects and 78 sampling plots in S1. The setting of belt transects in S2 was on north side of WEGP II by the same method. Considering only WEGP II passed through S2, no sampling plots were set between the two pipelines (Fig. 10B). Hence there were 9 belt transects and 54 sampling plots in S2 for vegetation investigation. The working area, which was also called the pipeline right-of-way, comprised the trench for the pipeline, a soil stacking area, and a lane (companion road) (Fig. 11). Six sampling plots were selected as CK in 2000 m away from the edge of northernmost pipeline area.

The types of species and numbers of plants were assessed in each sampling plot. The Percent Cover of Vegetation was visually estimated by three appointed persons. Then, an average value and the total coverage were calculated. Photographs were taken at each plot to calibrate the results of visual estimation. The vegetation investigation was conducted in July and August 2013, during the rainy season. The shrub *Tamarix chinensis* Lour. was not selected when setting out sampling plots because there were few examples of this shrub species and they usually had a large crown width that could fill the whole sampling plot. This species would have a high importance value if it occurred in a sampling plot, and the results would be skewed.

The importance value of each species was calculated as follows (Eqs 1–4):

1 $$IV=(Rd+Rf+Rc)/3$$

2 $$Rd=d/D$$

3 $$Rf=f/F$$

4 $$Rc=c/C$$

where *IV* is the importance value; *Rd* is the relative density; *Rf* is the relative frequency; and *Rc* is the relative coverage; *d* is the count of stalks of one species in unit area; *D* is the total stalks in unit area; *f* is the frequency of occurrence in the six plots in a belt transect; *F* is the sum of the *f*s of all the species found in the belt transect; *c* is the percent cover of vegetation of the species in a plot; C is the total percent cover of vegetation in a plot. Relative coverage was used to represent the relative dominance of each species. The integrated importance value (IIV) was introduced to represent the total importance values of the plant species that were classified in one group by cluster analysis. The IIV of each cluster was calculated as the arithmetic mean of importance values of all species in that cluster. And the IIV of G was the sum of the IIVs of clusters in that group. Considering the classified group would be estimated by the position in the community in the next step, IIV was a good choice. Firstly, given that IIV was the arithmetic mean of IVs of a group, and the IV represented the position of one species in a community, the IIV also represented the position of a group in the community that exactly met the requirement. Secondly, it was a relative value in a community, and therefore it was convenient to be compared with that of another group. Hence, IIVs were selected to reflect the position of the groups.

The selected vegetation indices used to reflect the vegetation condition of affected zones were the Shannon-Wiener diversity index (H), Pielou’s evenness index (Evenness), Margalef’s richness index (Richness) and Percent Cover of Vegetation. Evenness represents the uniformity and Richness indicates the number of species in selected sampling plot. The value of these indices were set using Eqs 5–7:

5 $${H}=-\sum _{{i}={1}}^{{S}}[({ni}/{N})\,{\rm{lg}}({ni}/{N})]$$

6 $${Evenness}=-\sum _{{i}={1}}^{{S}}[({ni}/{N})\,{\rm{lg}}({ni}/{N})]/\mathrm{ln}\,{S}$$

7 $$Richness=(S-{1})/\mathrm{ln}\,N$$

where *i* = 1, 2, …*S*, which stands for all of the species in a plot; *S* is the number of species; *N* is the sum of the numbers of all species in a plot; *n*_*i*_ is the number of the *i*th species in a plot; *c* is the number of species that appear in both a plot and control area; and *a* and *b* are the number of species that appear in the plot and the control area, respectively.

### TWINSPAN classification

TWINSPAN (two-way indicator species analysis) is widely applied to investigate vegetation composition^43–45^. It was developed by M. O. Hill^46^ and is included in software packages for ecological data analysis, such as PC-ORD^47^. The TWINSPAN algorithm begins with primary ordination of sites along the first axis of correspondence analysis (CA)^48^. Then, the sites are divided into two clusters by splitting the first CA axis near its middle. Site classification is refined using a discriminant function that emphasizes species preferential to one or the other half of the dichotomy. The division process is then repeated until each cluster has no more than a chosen minimum number of quadrats^45, 49^. In this study, Roleček’s modified TWINSPAN^45^ was used because its clustering strategy was based on reciprocal averaging and the indicator values of indicator species. These features conformed to the requirement that classified a type of plant species to represent the extent and intensity of the linear construction. Three cluster groups were determined at the first and the second break. Then we calculated their IIVs and defined them as G1, G2, and G3 per variation of IIVs changed by the distance to construction. TWINSPAN was calculated by PC-ORD 5.0.
